# 3D Scanners in Orthodontics—Current Knowledge and Future Perspectives—A Systematic Review

**DOI:** 10.3390/ijerph18031121

**Published:** 2021-01-27

**Authors:** Maciej Jedliński, Marta Mazur, Katarzyna Grocholewicz, Joanna Janiszewska-Olszowska

**Affiliations:** 1 Department of Interdisciplinary Dentistry, Pomeranian Medical University in Szczecin, 70-111 Szczecin, Poland; maciej.jedlinski@pum.edu.pl (M.J.); katgro@pum.edu.pl (K.G.); 2 Department of Dental and Maxillofacial Sciences, Sapienza University of Rome, 00161 Rome, Italy; marta.mazur@uniroma1.it

**Keywords:** intraoral scanners, efficiency, accuracy, orthodontics

## Abstract

*Background*: Nowadays the use of intraoral scanners has become a routine practice in orthodontics. It allows the introduction of many treatment innovations. One should consider to what extent intraoral scanners have influenced the everyday orthodontic practice and in what direction should the further research in this field be conducted. This study is aimed to systematically review and synthesize available controlled trials investigating the accuracy and efficacy of intraoral scanners for orthodontic purpose to provide clinically useful information and to direct further research in this field. *Methods*: A literature search of free text and MeSH terms was performed by using MedLine (PubMed), Scopus, Web of Science and Embase. The search engines were used to find studies on application of intraoral scanners in orthodontics (from 1950 to 30 September 2020). The following keywords were used: “intraoral scanners AND efficiency AND accuracy AND orthodontics”. *Results*: The number of potential identified articles was 71, including 61 from PubMed, two from Scopus, three from Web of Science and five from Embase. After removal of duplicates, 67 full-text articles were analyzed for inclusion criteria, 16 of them were selected and finally included in the qualitative synthesis. *Conclusions*: There are plenty of data available on accuracy and efficacy of different scanners. Scanners of the same generation from different manufacturers have almost identical accuracy. This is the reason why future similar research will not introduce much to the orthodontics. The challenge for the coming years is to find new applications of digital impressions in the orthodontic practice.

## 1. Introduction

The first intraoral scanner—named CEREC—was developed by Dentsply Sirona (Charlotte, NC, USA) and came into production in 1985. Its purpose was to take the “impression” in order to design fixed prosthetic restorations [1,2]. In 1999 Orthocad (Czestochowa, Poland) introduced the first version of their software, which allowed the study of digital versions of orthodontic casts after proper scanning at the company headquarters, what can be considered as the introduction of 3D models into orthodontics [3]. Since then scanners have improved significantly, providing high quality mapping of both hard and soft tissues, being able to replace traditional plaster models [4]. The inconvenience of pouring and trimming plaster casts as well as the need to take them out of storage on every visit is no longer a must. Nowadays one can view teeth on the computer screen and manipulate them freely in 3D on different devices, which further facilitates communication with a patient [5]. The question asked in the revision on orthodontic scanners from 2015 by Martin et al. “Will intraoral scanners be routinely used in orthodontics?” [3] should be answered in the affirmative. Since then plenty of data in literature are available in which the authors deal with the introduction of scanners in everyday dental practice on the basis of various specialties—prosthodontics, dental surgery or orthodontics. Moreover, the same authors pointed out that intraoral scanners made it possible to introduce innovations in orthodontics such as monitoring dental movement through digital model superimposition [6], aligners [7], further customization of orthodontic appliances such as removable retainers [7] and last but not least, more accurate diagnosis, treatment planning and even simulation of possible orthodontic movement on appropriate software [8,9]. However, one should ask a question to what extent intraoral scanners have influenced the everyday orthodontic practice and in what direction should the further research be conducted to make this technology even more useful for clinicians.

The aim of this study was to systematically review and synthesize available controlled trials investigating the efficiency and accuracy of orthodontic intraoral scanners in order to provide useful information to make clinical decisions, and to direct further research in this field.

## 2. Materials and Methods

This systematic review was performed according to the PRISMA statement [10] and by following the guidelines from the Cochrane Handbook for Systematic Reviews of Interventions [11]. The framework of this systematic review according to PICO [12] was: Population: orthodontic patients; Intervention: scanning oral cavity; Comparison: traditional impressions or no intervention; Outcomes: efficiency and accuracy.

### 2.1. Search Strategy

**Figure 1:**
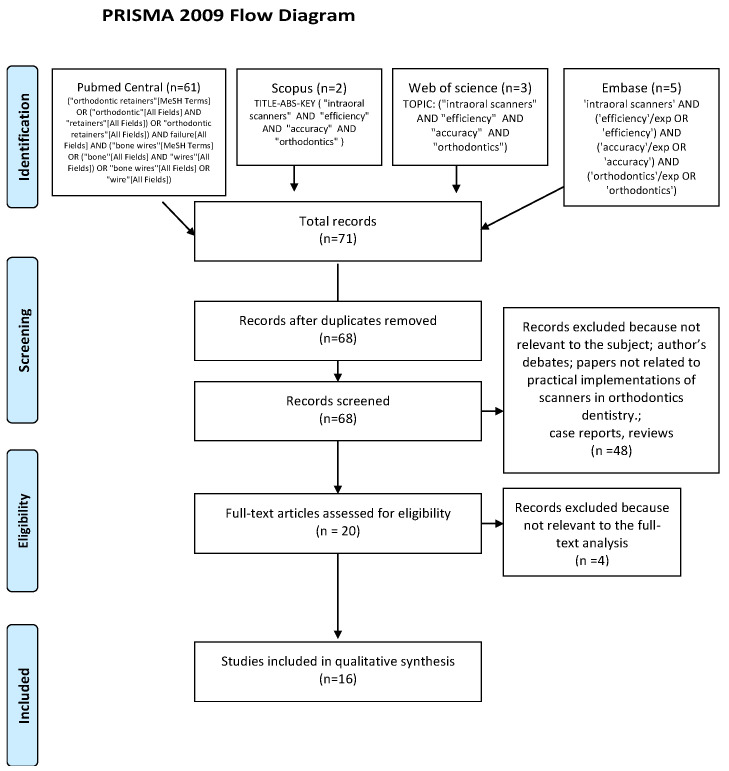
Prisma Flow Diagram.

Literature searches of free text and MeSH terms were performed by using MedLine (PubMed), Scopus, Web of Science and Embase (covering from 1950 to 30 September 2020). All searching was performed using a combination of subject headings and free-text terms: we determined the final search strategy through several pre-searches. The keywords used in the search strategy were as follows: (“intraoral scanners AND efficiency AND accuracy AND orthodontics”). Search strategy for MedLine (PubMed Central), Scopus, Web of Science and Embase is presented in Figure 1. Reference lists of primary research reports were cross-checked in an attempt to identify additional studies. 

### 2.2. Eligibility Criteria

The following inclusion criteria were employed for this systematic review: (1) randomized clinical trial (RCT); (2) cohort study; (3) case-control study; (4) articles in the last five years (5) published in English; all the potentially evaluated articles were supposed to explore the subject of development, accuracy and innovatory ways of using scanners in orthodontics. The following exclusion criteria were applied: (1) case reports; (2) reviews; (3) abstract and author debates or editorials; (4) lack of effective statistical analysis; (5) papers not related to practical implementations of scanners in orthodontics or dentistry.

### 2.3. Data Extraction

Titles and abstracts were independently selected by two authors (M.J. and J.J.-O.), following the inclusion criteria. The full text of each identified article was then analyzed to verify whether it was suitable for inclusion. Disagreements were resolved through consensus or by discussion with the third author (M.M.). Authorship, year of publication, type of each eligible study and its relevance regarding the use of scanners in everyday practice were independently extracted by two authors (M.M. and M.J.) and examined by the third author (J.J.-O). Characteristics of the studies included have been presented in Table 1.

### 2.4. Quality Assessment

According to the PRISMA statements the evaluation of methodological quality gives an indication of the strength of evidence provided by the study, because methodological flaws can result in biases [10].

The quality assessment was performed using Jadad scale for randomized controlled trials for RCT and RCCT studies [13]. It was taken into account in the assessment whether the study was randomized, double-blind with appropriately described methods to find out the level of the risk of bias. A point was given for every characteristic evaluated, when the possible assessment was from zero to five, with a high score indicating a good quality of a study. Notwithstanding, for Case-control Studies, the Newcastle-Ottawa Quality Assessment Form [14] was used. The quality of all included case-control studies was based on object selection, comparability, and exposure. The possible quality assessment score ranged from zero to nine points with a high score indicating a good quality study. There was one point awarded for each characteristic evaluated.

## 3. Results

### 3.1. Study Selection

The search strategy identified 71 potential articles: 61 from PubMed Central, two from Scopus, three from Web of Science and five from Embase. After removal of duplicates, 67 articles were analyzed. Subsequently, 48 papers were excluded because they did not meet the inclusion criteria. Of the remaining 20 papers, four more were excluded because they were not relevant to the subject of the study. The remaining 16 papers were included in the qualitative synthesis. A Prisma 2009 flow diagram representing the study selection process has been presented in Figure 1.

Table 1 summarizes the characteristics of each of the 16 included studies.

It should also be noted that: (i) five of all included studies focused on comparison of the accuracy and clinical effectiveness of different scanners in relation to one another; (ii) seven studies tried to implement the innovative methods of the use of scanners in the daily work of dental practice. Moreover, five of them focused on both checking accuracy of the scanners and implementing the novelties.

The studies included in this review used a large variety of scanners:

- 3Shape (n studies: 12; 3Shape Trios -n 7- and 3Shape TriPod -n 5-);
- iTero Element (n studies: 4);
- Carestream 3600 (n studies: 4);
- OrthoinSight 3D*-extraoral scanner (n studies: 3)
- Other (n studies: 5)

### 3.2. Quality Assessment and the Risk of Bias

Quality assessment is shown in Table 2 for the randomized controlled trials (RCTs) and in Table 3 for the case-control studies.

According to the Jadad scale for RCT, the authors evaluated the qualities of all four clinical trials included in the qualitative synthesis, based on five questions that analyze the randomization process, the experimental blinding and the appropriate time of follow-up. In the evaluation of the quality of RCTs, the total score of three studies was equal to 5, indicating high-quality studies [17,19,26]; while one scored 3, indicating a low-quality study [15]. Blinding is present in three RCT studies (Yilmaz et al. [26] Nalaci et al. [17] and Kim and Lagravére [19], Table 2).

According to the Newcastle–Ottawa scale, the authors evaluated the qualities of all 12 case-control studies included in the qualitative synthesis, based on object selection, comparability, and exposure. In the evaluation of the quality of case-control studies, the total score of eight studies was 9, indicating high-quality studies [15,18,20,21,24,27,28,29]. Then two studies scored 6 [25,30], indicating a medium quality; one 7 [23] and one 8 [22] (Table 3).

## 4. Discussion

This systematic review endeavored to comprehensively display the available evidence on the efficiency and accuracy of scanners used in orthodontics, in order to document the state of the art and further development.

A total of 16 studies were included in this review, four RCTs and 12 case-control studies. Different scanners were analyzed: 3Shape Trios (3Shape, Copenhagen, Denmark) in twelve studies, iTero Element (Aligntech, San Jose, CA, USA) in four studies, Carestream 3600 (Carestream, Rochester, NY, USA) in four studies, OrthoinSight 3D* (Motion View Software, LLC, Chattanooga, TN, USA) in three studies, Lavacos (3M, Maplewood, MN, USA) in two studies. 

The included studies were critically revised and they focused mainly on: (i) determination of the accuracy of scanning and measurement on digital models of different scanning devices; (ii) implementation of the innovative approaches in the use of intraoral scanners. 

### 4.1. Determining the Accuracy of Scanning and Measurement on Digital Models of Different Scanning Devices

In total there were 10 papers analyzing the accuracy of scanners and measurement on digital casts and their comparability to plaster models and measurements on them [19,22,23,26,30], to models processed by high-efficiency industrial scanners [21,27], evaluation of accuracy of one scanning device in comparison to another [15,20,22,27,28]. All authors underlined that the accuracy of intraoral scans allowed them to replace classic dental models, as the quality of tissue mapping is the same or better than in the classical method [20,23,26,27]. Interestingly, there were no significant differences in the mapping of the oral cavity between the direct and indirect technique, which eliminates the necessity of casting plaster models [21,24]. Furthermore, scanning requires more chairside time, but it was found less unpleasant than standard procedure of impression taking [21]. However, evidence exists that patients when asked which type of impression satisfy them more, choose digital, due to patient-centered outcomes [31]. Some authors pointed out that the results of the studies, which showed the differences in accuracy between different intraoral scanners models, could have significant impact for future research, but the differences in tissue mapping between different models did not significantly alter the clinical evaluation of the orthodontic patient [20,21,22,27]. Solabrietta et al. pointed out that the differences in accuracy between the scanners are rudimentary, and the characteristics that make every daywork easier and more enjoyable for the doctor and the patient seem to be much more important. As example, Lava Cos and Trios 3-Shape intraoral scanners showed similar characteristics, both of them being good enough to carry out needed procedures, but LavaCos requires coating and had a comfortable small tip, whereas the Trios 3-Shape scanner had a larger tip and worked much faster [20]. In the study of Jacob et al. all, scanners used in the study (iTero element, Lythos, Ortho Insight 3D) showed similar and reliable measures. However, the most errors were found in the Ortho Insight 3D measurements, as on its arch length and canine height were systematically underestimated [15]. One must remember that this scanner does not shorten the working time as it does not allow one to avoid the impression-taking procedure. 

It is easy to behold that the topic of comparing the accuracy of digital models is extremely popular and has been present in the literature for so long that separate systematic reviews have been developed about it [4]. The results of included studies lead to the general statement that for clinical purposes, deviations in the accuracy between the different scanners are irrelevant [15,20,22,27,28]. Naturally, new papers are constantly being published, however, they focus more often on implantology, where accuracy on smaller orders of magnitude can make a difference [32,33]. For the purposes of orthodontists, however, until a new generation of products hits the market, further studies seem redundant, and the accuracy of current devices seems to be at least satisfactory.

The prices of scanners of different manufacturers are comparable. However, extraoral scanners seem to be a cheaper alternative to intraoral scanners when used for longer period of time. The 3Shape Trios has been marketed since 2015. Its average cost is about $35,000 for the current version scanner and $3500/year for a subscription to the dedicated PC software. The iTero Element is marketed since 2015 with a cost of $28,000 for the current version scanner and $4320/year for a subscription to the dedicated PC software. The Carestream CS 3600 is marketed since 2016 at a cost of $40,000 for the scanner and $2,200$/year for a subscription to dedicated PC software. Currently 3M (St. Paul, MN, USA) offers the True Definition scanner for $17,000$ for the current version scanner and $230/year for a subscription to dedicated PC software.

3Shape Tripods are marketed since 2012 at a cost of $31,000 for the current version of the scanner and $1900 for a subscription to the dedicated PC software. The OrthoinSight 3D is marketed since 2012. Its price, however, is not openly available either on the manufacturer’s website or at any local provider.

Other factors, which should affect the choice of proper scanner, are the consistency of software and solutions proposed by the producer. One should pay attention to the cooperation between the office and the final user of the scan—the dental technician.

### 4.2. The Innovative Approaches in Use of Intraoral Scanners

The presence of intraoral scanners in orthodontic practice has already revolutionized many types of treatment. When treatment with Invisalign’s transparent aligners was introduced in 1997, laser scans were approached with caution [34]. It involved converting PVS impressions to computer scans at the company’s premises [35]. After the iTero scanners were launched on the market and the physician-technician communication was transferred to the Clinchek platform, access to this technology was facilitated, treatment planning and monitoring was improved, and its efficiency increased [36]. Is there a chance for an innovative proposal, thanks to which scanners will once again change orthodontic practices? In the remaining 11 included papers, authors proposed some innovations in the use of intraoral scanners. 

Among them there should be distinguished:

(a) New improvements in general use
  - proposing a new training to maximize the efficiency of dental hygienist [16]
  - orthodontic movement monitoring in quicker and much more comfortable way than before [17]
  - finding that full intraoral scans are perfectly reliable for orthodontic cases, but still not useful for prosthetic cases, where up to 3 segments should be scanned [21]
  - using the digital scans as exact for documentation of palatal soft tissue [23]
  - the innovative method of scanning the palate, ensuring its most faithfully reproduction on the digital model [24]
  - finding that when clinician depend on digital models, it is best to use ceramic brackets as they provide the lowest discrepancy of measurements [28]
  - finding that scanning method, which provides most accurate digital casts is when scanning begins from tooth #12 up to tooth #17, and then from tooth #12 up to tooth #27 [25]

In order to monitor orthodontic treatment, the standard is the periodic repetition of radiological examinations such as a panoramic radiograph or cephalogram, what is evidenced by the methods used in many recently published studies [37,38]. However, the radiation of patients should be minimized, especially during their period of growth [39]. On the other hand, comparing the measurements performed on the model and in the patient’s oral cavity in vivo may be burdened with considerable operator error [40]. Scanners seem to be an ideal, accurate and safe alternative in this case. It is encouraging that clinicians appreciate a feature that only scanners can provide, i.e., assessments of soft tissue features such as exact shape, which changes gradually throughout the treatment, [24] and color, saturation or swelling [21,23]. It is also interesting that the type of brackets is quite important for the fidelity of turning the tooth surface through the scanner [28]. It is such studies, with specific clinical recommendations, that can spread the use of scanners and increase their efficiency.

(b) Completely new applications in the use of scanners
  - use *rugae palatine* patterns on digital scans in order to identify individuals [18]
  - determine the occlusal contacts in a novel way [20]
  - display in more accurate way the interdental areas in periodontal patients directly in patients’ mouth [29]
  - finding centric relation only by using intraoral scanner [30]

Moreover, an increasing trend is to treat patients centrally, which, according to many doctors, ensures greater stability of treatment [41]. The growing popularity of courses in line with the Functional and Cosmetic Excellence (FACE) philosophy is not without significance [42]. Currently, accurate determination of occlusal contacts and the articulation of the patient are a tedious process that often requires a team of many people and the help of a technician. The proposed methods could significantly shorten the patient’s articulation process and, secondly, make it more common [20,30]. Interestingly, intraoral scanner was used to identify people [18] and it shows the skill of scanners to carefully assess the palatal soft tissue, proven by Deferm et al. [23] Assessment of the condition of the palate is an important part of monitoring the progress of orthodontic treatment as well as monitoring the interdental areas in periodontal patients during orthodontic therapy [29]. These are the new and interesting proposals, that the world of scanners needs to keep growing.

## 5. Conclusions

In conclusion, included studies focused on scanners’ development and implementation to solve many challenging elements of orthodontic therapy. On the other hand, there are plenty of data available on accuracy and efficacy as well as mapping comparisons of different scanners. Scanners of the same generation from different manufacturers have almost identical accuracy. This is the reason why similar research will not introduce much to the orthodontic case evaluation or treatment. Any new reports should focus solely on comparing the new, untested models. Scanners are a modern, adequate and increasingly accessible source for capturing and imaging the appearance of oral tissues. The challenge for the coming years is to find new applications of digital impressions and imagining in the orthodontic practice. In the upcoming studies scanners should serve only as a tool for observing clinical phenomena.

## Figures and Tables

**Table 1:** Characteristics of the included studies.

Author, Year [Reference]	Type of Study	Study Objective	Study Material	Number of Subjects	Test Group	Control Group	Intraoral Scanner Model	Results
Jacob et al. 2015 [15]	Case—control study	Comparison of the accuracy of two intraoral scanners and one extraoral scanner	3D intraoral and extraoral scans	15 subjects	Intraoral scans, extraoral scans of dental cats	Intraoral scans, extraoral scans of dental cats	iTero element, Lythos Ortho Insight 3D,	While all three scanners produced reliable measurements, Ortho Insight 3D systematically underestimated arch length and canine height. Measurements taken from all three scanners were highly reliable, with intraclass correlations ranging from 0.926 to 0.999. Method errors were all less than 0.25 mm
Park et al. 2015 [16]	RCCT	Evaluation of the influence of proper training in intraoral scanning among dental hygienists.	Questionnaires before and after the training	34 hygienists, 17 per group. The hygienists from the other group served as patients in the group using a different scanner, thus assessing the patient’s comfort during the scanning.	iTero element group	3Shape group	iTero element, 3Shape Trios	The first preliminary questionnaire was given initially before the training sessions and the second questionnaire was completed upon the completion of all the training sessions. Both evaluated parameters as difficulties of using intraoral scanners with digital impression method compared to conventional impression-taking method, patient comfort, and degree of awareness about intraoral scanners. The parameter of awareness included anticipated accuracy, patient convenience, efficiency, clinical application, and interest in further use. Results of this study indicated that participants generally preferred Trios intraoral scanner over iTero as an operator after the training. However, participants consider iTero as easier to handle during the scan and indicated, that it provides much higher patients comfort. The usefulness of intraoral scanner could be a successful alternative to conventional impression-taking with proper training.
Nalaci et al. 2015 [17]	RCCT	Evaluation of reliability of measurements by superimposition of 3D digital models	Digital models, classic dental casts and cephalometric radiographs of adolescent patients	10 female and 10 male II Class subjects; (mean age: 16)	The posterior movements of the maxillary first molars evaluated on superimposed 3D models before and after treatment	(a) The distal movements of the maxillary first molars evaluated on cephalometric radiographs (b) The distal movements of the maxillary first molars evaluated on photocopies of plaster models	3 Shape R700 model	There was no significant difference observed in values of distalization of the upper molars, premolars, or incisors among all three groups. The measurement differences among the 3D digital models, cephalometric radiography, and plaster model photocopy methods were insignificant. Cronbach’s alpha value was closest to ideal 1 for the measurement on 3D models. The use of superimposed 3D models on Orthocad Software as the evaluation of treatment results seems as valid alternative for conventional evaluation method.
Taneva et al. 2015 [18]	Case—control study	Creation of control points on *rugae palatine* patterns using 3D digital models in order to identify victims	3D intraoral scans, 2D photos	15 subjects from the university clinic and 15 subjects from private orthodontic practices	Intraoral scans, extraoral scans of dental cats	Scans after follow-up, 2D photos	iTero element, Ortho Insight 3D	No statistically significant mean differences exist between different digital model conversion techniques, between OrthoCAD™ and Ortho Insight 3D™, and between Ortho Insight 3D™. Twelve palatal 3D landmarks could be used for human identification over time, certain landmarks showed more significant impact on the matching process
Kim and Lagravére 2016 [19]	RCCT	Comparison of accuracy of 3D models of conventional dental casts vs. CBCT scans	Digital models, classic dental casts and CBCT scans of adolescent patients	50 models and corresponding casts and digital models	Measurements on the 3D models using Ortho Insight 3D software	(a) Measurements of CBCT scans converted to DICOM files using Avizo Software (b) Manual measurements taken on classic dental cast	Ortho Insight 3D laser scanner	Intra-examiner measurement errors were determined by randomly selecting 10 patient records, and the mesiodistal width measurements were repeated three times 1 week apart by the same examiner. CBCT exhibited the lowest error reliability, while scanned digital models had the highest intra-examiner error reliability. The mutual compatibility of measurements deviates in the case of calculating anterior Bolton ratio at the level of agreement of 0.886. However, Bolton analysis can be accurately and reliably performed on scanned digital models using the proposed Ortho Insight system.
Solaberrieta et al. 2016 [20]	Case—control	To locate the 3D spatial position of the mandibular cast and determine its occlusal contacts in a novel way by using an intraoral scanner as part of the virtual occlusal record procedure	3D intraoral scans, dental casts	4 participants	3D intraoral scans	3D scans from industrial scanner	Lava Cos, 3Shape Trios, Zfx Intrascan, ATOS Compact Scan 5M	Intraoral virtual occlusal recording is a valid procedure to locate a mandibular cast on a virtual articulator. The contacts observed with this procedure were accurate enough. Moreover, virtual contacts provided more objective and meaningful information. Lava Cos and Trios 3-Shape intraoral scanners showed similar characteristics, both of them being good enough to carry out this procedure. The LavaCos requires coating and has a comfortable small tip, whereas the Trios 3-Shape scanner has a larger tip and is much faster. The best results regarding the virtual occlusal record sections were obtained when the distance between the sections was maximum. Two or three sections can be required to establish perfect occlusal record.
Wesemann et al. 2017 [21]	Case—control compa-rative study	Comparison of the accuracy and time efficiency of an indirect and direct digitalization workflow with that of a 3D printer to identify the most suitable method for orthodontic use.	3D intraoral scans, CBCT scans, conventional dental casts	None—comparison of the scanning quality on hypothetical dental cast	Intraoral scans of master model with separately: 3Shape R700, 3Shpae R900, 3Shape Colorpod	Dental casts, CBCT scans	3 Shape Tripod R700 3Shape Tripod R900 3Sape Trios Colorpod	The most accurate results were obtained by the R900. The R700 and the TRIOS intraoral scanner showed worse, but still comparable results. CBCT-3D-rendering revealed significantly higher accuracy with regard to dental casts than dental impressions. The chairside time required for digital impressions was 27% longer than for conventional impressions. For orthodontic demands, intraoral scanners are a useful alternative. For prosthodontic use, no more than one quadrant and three additional teeth should be provided as the scanning area on one seat.
Lee 2018 [22]	Case—control study	Comparison of two intraoral scanners based on 3D surface analysis	3D intraoral scans and dental casts scans	32 adult participants	3D intraoral scans	3D dental casts scans	3Shape Trios 3 iTero Element 2	The mean deviations between two intraoral scans were 0.057 mm in the maxilla and 0.069 mm in the mandible. The histogram showed local variations between the two scanners in the posterior area. In three-dimensional deviations, intraoral scans showed a slight shift towards the models. There were no statistically significant differences between the two scanners.
Deferm et al. 2017 [23]	Case—control study	Assessment of the feasibility of 3D intraoral scanning for documentation of palatal soft tissue by evaluating the accuracy of shape, color, and curvature.	3D intraoral scans, dental casts	Scans of 10 participants by 2 different observers	Intraoral scans	Dental casts	3Shape Trios	Mean average distance error between the conventional models and the intraoral scans models was 0.02 ± 0.07 mm. Mean interobserver color difference was −0.08 ± 1.49°, 0.28 ± 0.78% and 0.30 ± 1.14% for respectively hue, saturation, and value, whilethe interobserver differences for overall and maximum surface irregularity were 0.01 ± 0.03 and 0.00 ± 0.05 mm. what showed that intraoral scanner is tool, which provide reliable, reproducible and accurate results, and therefore allows to freely document the palate.
Zhonpeng et al. 2019 [24]	Case—control study	Comparison of the differences in palatal region between indirect and direct digital models and the scanning sequences	3D intraoral scans and dental casts scans	35 adult participants (9 men and 26 women (24–27 years old)	(a) 3D intraoral scans with the start point behind the central incisors (b) 3D intraoral scans with the start point behind the central incisors	Scans of dental casts	3Shape Trios 3 Pod	When evaluating accuracy of intraoral scanning in the palatal region, the superimposition method should be appropriately adjusted. It means that to obtain a certain comparison of scanning accuracy, soft tissues (palate), dentition (dental arch) and the entire scan (angulation of one to the other) must be assessed in separated evaluation. Palatal trueness is affected by scanning sequences. To get the best impression one needs to begin the scanning procedure from the palatal side of posterior teeth.
Favero et al. 2019 [25]	Case—control study	Comparison of the accuracy of 3D digital impressions obtained using two intraoral scanners and three scanning methodologies.	3D intraoral scans	None—comparison of the scanning quality on hypothetical dental cast	Technique A (from tooth #27 up to tooth #17); Technique B (from tooth #11 up to tooth #17 and then from tooth #21 up to tooth #27) Technique C (from tooth #12 up to tooth #17, and then from tooth #12 up to tooth #27) - scanned with Carestream 3600	Technique A (from tooth #27 up to tooth #17); Technique B (from tooth #11 up to tooth #17 and then from tooth #21 up to tooth #27) Technique C (from tooth #22 up to tooth #17, and then from tooth #12 up to tooth #27) - scanned with Zfx Evolution	Zfx Evolution, Carestream 3600	The scanning technique had a statistically significant effect on the quality of the scan (*p* < 0.0001).When scanning begins from tooth #12 up to tooth #17, and then from tooth #12 up to tooth #27 it is less volumetric whereas the scanner did not present any significant influence (*p* = 0.91).
Yilmaz et al. 2019 [26]	RCCT	Comparison of Accuracy of 3D models vs. conventional dental casts	Digital and classic dental casts of adolescent patients	20 female, 10 male subjects; (mean age: 14.36 ± 6.30)	Measurements on 3D models with 3Shape Ortho Analyzer 2013 software	Manual measurements on classic dental cast	3 Shape TriosColor-P1	The study checked the accuracy of measurements of the spaces between the teeth in both dental arches and also Bolton’s analysis was carried out. To increase the reliability of the measurements, they were repeated five times, and the arithmetic average value was used for the evaluation. Conventional analysis required 33% more time then the digital one. (mean 894.33 s vs. 597.73 s) There were no significant differences between the accuracy achieved *p* < 0.001. The digital analysis is as reliable as conventional model analysis and it seems to be more time effective.
Winkler & Gkantidis 2020 [27]	Case—control study	Comparison of the precision of intraoral scanners and the industrial scanner	3D scan with industrial scanner Artec Space Spider, with CS 3600, with TRIOS 3	12 adult volunteers (8 men, 4 women, age: 27–52 years)	3D scans with the use of: (a) CS 3600, (b) TRIOS 3.	3D scan with industrial scanner Artec Space Spider	Carestream 3600 3Shape Trios 3	The precision of the intraoral scanners (TRIOS 3 and CS 3600) was tested after superimposing the dental arch surface models obtained from repeated scans. In overall, TRIOS 3 showed better precision than the CS 3600(approximately 10μm). However, looking into the detail, both devices showed a clear imprecision, especially on buccal side of anterior teeth. Both devices do not show the accuracy of an industrial scanner, however, their accuracy is completely sufficient for clinical applications.
Jihu Song & Minji Kim 2020 [28]	Case—control study	Evaluation of scanned images of 4 clinically used intraoral scanners with different types of brackets	3D intraoral scans	4 different study models	(a) Study model with metal brackets (b) Study model with ceramic brackets (c) Study model with resin brackets	Study model without any brackets	Carestream CS3600, i500, 3Shape Trios3, Sirona Omnicam	Image data were analyzed with the 3D analysis software—Geomagic. Not only both scanners (*p* < 0.05) and brackets (*p* < 0.05) had a significant impact, but also interaction between scanner and brackets was statistically significant (*p* < 0.05). Brackets which were translucent or reflective to light such as resin and metal brackets tended to show higher discrepancy values. From the point of view of the clinician, all scanners showed acceptable deviation results, however CS3600 and Trios3 were most accurate comparing to control model.
Schlenz et al. 2020 [29]	Case—control study	Analysis of the ability of analog and digital impression techniques to display the interdental areas in periodontal patients	3D scans and dental casts	30 patients, age: 48–87	Intraoral scans	Conventional dental casts	True Definition, Carestream 3600, 3Shape Trios 3, Primescan	True definition scanner gave the most adequate results, displaying the highest percentage of interdental areas, followed by Primescan, Carestream and 3Shape. However, regardless the technique of scanning or type of scanner, intraoral scans always demonstrated the number and condition of recessions better than conventional casts.
Stafeev et al. 2020 [30]	Case—control study	Comparison of classic method of finding centric relation to new digital method using 3D Scans	3D scans and classic models and frontal deprogrammer	5 patients, 20 registrations of the centric jaw relation for each patient	Intraoral scans	Conventional deprogrammation method	3Shape Trios 3	The reproducibility of the digital mandible position in the centric relation reached 0.119 ± 0.012 mm for frontal deprogrammer, 0.225 ± 0.028, *p* ≤ 0.05 for bilateral manipulation by *p*.E. Dawson, 0.207 ± 0.02, *p* ≤ 0.05 for leaf gauge, and 0.120 ± 0.013, *p* ≤ 0.05 for intraoral device for recording the Gothic arch angle. All methods of searching for centric relation do not coincide completely. However, the most precise were the methods using the frontal deprogrammer and the intraoral recording of gothic angle. In terms of reproducibility, the Avantis 3D program most accurately identified the mandible position, when searching for the position of centric relation. By choice of the many, many factors that should be considered such as the condition of the stomatognathic system, the manual skills of the doctor, the psychoemotional status of the patient, and the material provision.

**Table 2:** Evaluation of included RCTs according to Jadad Scale.

Author	Park et al. 2015 [16]	Nalaci et al. 2015 [17]	Kim and Lagravére 2016 [19]	Yilmaz et al. 2019 [26]
Randomization present	1	1	1	1
Appropriate randomization used	1	1	1	1
Blinding present	0	1 *	1 *	1 *
Appropriate blinding used	0	1 *	1 *	1 *
Appropriate long-term follow-up for all patients	1	1 *	1 *	1 *
Total	3	5	5	5

* In case of studies comparing the accuracy of different methods, more than one measurement was conducted after a longer period of time.

**Table 3:** Evaluation of case—control studies according to Newcastle—Ottawa quality assessment.

**Study**		**Jacob 2015** [15]	**Taneva et al. 2015** [18]	**Solaberrieta et al. 2016** [20]	**Wesemann et al. 2017** [21]
**Selection**	Is the case definition adequate?	1	1	1	1
	Representativeness of the cases	1	1	1	1
	Selection of Controls	1	1	1	1
	Definition of Controls	1	1	1	1
**Comparability**	Comparability of cases and controls on the basis of the design or analysis	2	2	2	2
		The procedures of intraoral scanning are the same for both scanners. The choice of the extraoral scanner as a control is not objectionable. In the Ortho Insight, the mandibles were secured with double-sided tape. Each mandible was scanned twice, at least 1 week apart, using each of the three scanners. Scans were extracted in .stl format. Cases and control do not cause any doubt from methodological point of view.	To estimate the identification points on *rugae palatine* five cross sections in the anteroposterior dimension and four cross sections in the transverse dimension were computed which generated 18 2D variables. Both cases and control obtaining method are well described and do not cause any doubt from methodological point of view.	To make a control model, the teeth were scanned using Zfx Evolution -widely recognized as being a high precision reference scanner. Scans were extracted in .stl format. Both cases and control obtaining method are well described and do not cause any doubt from methodological point of view. Scans were extracted in .stl format.	The control model was measured with a coordinate measuring instrument. All the cases were comparable and measured with details to ensure comparability of time effectiveness and accuracy of indirect and direct workflow. Both cases and control obtaining method are well described and do not cause any doubt from methodological point of view. Scans were extracted in .stl format.
**Outcome**	Ascertainment of exposure	1	1	1	1
	Same method of ascertainment for cases and controls	1	1	1	1
	Non-Response rate	1—authors described whenever the scanner did not process the data perfectly	1—authors described whenever the scanner did not process the data perfectly	1—authors described whenever the scanner did not process the data perfectly	1—authors described whenever the scanner did not process the data perfectly
**Total**		9	9	9	9
**Study**		**Lee 2018** [20]	**Deferm et al. 2018** [21]	**Zhongpeng et al. 2019** [22]	**Favero et al. 2019** [23]
**Selection**	Is the case definition adequate?	1	1	1	1
	Representativeness of the cases	1	1	1	1
	Selection of Controls	1	1	1	1
	Definition of Controls	1	1	1	0
**Comparability**	Comparability of cases and controls on the basis of the design or analysis	2	1	2	1
		All intraoral scans with the iTero and TRIOS scanners were recorded by the same single examiner. The scanners were calibrated every 8 days according to the manufacturers’ recommendation. Both scans were performed in a predetermined sequence and analyzed in open .stl format in exterior software.	Although obtaining the scan of curvatures and distances of palatal region of the case and control group was well described, there was no special control color description, as hue, saturation, value were measured only on the case scans, as control was performed on classic cast. Scans were extracted in .stl format	Scanning in both sequences was performed with the device by experienced operators. The way the digital and classical casts were obtained is well described and did not influence the shape of the palatal folds to ensure comparability of accuracy of indirect and direct workflow. Scans were extracted in .stl format. Both cases and control obtaining methods are well described and do not cause any doubt from methodological point of view.	To make a control model, the teeth were scanned using Zfx Evolution—widely recognized as being a high precision reference scanner. However, the method of obtaining the control model was not precisely described, citing only that the laboratory that made it is highly respected. In a study comparing the effect of scanning sequences, it would be useful to explain the process of creating an image serving as a reference. Not in all of the sequences it has been described if the sequence was started from buccal or palatal side of element.
**Outcome**	Ascertainment of exposure	1	1	1	1
	Same method of ascertainment for cases and controls	1	1	1	1
	Non-Response rate	No description	1—authors described whenever the scanner did not process the data perfectly	1—authors described whenever the scanner did not process the data perfectly	No description
**Total**		8	7	9	6
**Study**		**Jihu Song & Minji Kim 2020** [27]	**Winkler &Gkantidis 2020** [28]	**Schlenz et al. 2020** [29]	**Stafeev et al. 2020** [30]
**Selection**	Is the case definition adequate?	1	1	1	1
	Representativeness of the cases	1	1	1	0
	Selection of Controls	1	1	1	1
	Definition of Controls	1	1	1	1
**Comparability**	Comparability of cases and controls on the basis of the design or analysis	2	2	2	1
		Every model was bonded with ceramic, metal, and resin brackets, respectively, and without brackets. Reference images were taken by scanning the models with an industrial scanner. Then artificial saliva was applied on study models and model was scanned 10 times, respectively, with 4 different intraoral scanners. All images were converted to STL file format and analyzed with 3D analysis software.	To make a control model, the teeth were scanned using Artec Space Spider-recognized as being a high precision industrial scanner. Scans were extracted in .stl format. The procedures of intraoral scanning were the same for both scanners. What is more, every tooth was divided in segment and the precision and thruthness analysis is performed. The procedures of intraoral scanning are the same in both scanners. Both cases and control do not cause any doubt from methodological point of view.	Cases were performed with intraoral scanners following manufacturers’ recommendation. A classic dental cast was used as control. Both cases and control obtaining method are well described and do not cause any doubt from methodological point of view. Scans were extracted in .stl format.	The authors tried to do the research of innovative nature, as searching for the centric relation in “virtual articulator” there are too many factors influencing the actual centric relation of the patient and the proper interpretation of actual centric relation by the program, that the representativeness of the cases remains questionable. We do not know, wherever all the effects of deprogrammation were similar in all patients as the software described. Despite all of that, research results seem promising.
**Outcome**	Ascertainment of exposure	1	1	1	1
	Same method of ascertainment for cases and controls	1	1	1	1
	Non-Response rate	1—authors described whenever the scanner did not process the data perfectly	1—authors described whenever the scanner did not process the data perfectly	1—authors described whenever the scanner did not process the data perfectly	No proper description
**Total**		9	9	9	6

## Abbreviations

RCT	randomized control trial
RCCT	randomized control clinical trial
3D	three dimensional;
CT	computed tomography
